# Supplementary material for: The potential role of genetic assimilation during maize domestication
Source: PLoS One. 2017 Sep 8;12(9):e0184202. doi: 10.1371/journal.pone.0184202 (PMC5590903; doi:10.1371/journal.pone.0184202)
Supplement: S1 Table — (PDF) [file pone.0184202.s001.pdf]

Table S1. Sources of the teosinte and maize seeds.

USDA accession ID	Lat/Lon	Origin
PI 384062, pop1	17.417 N, -99.5 W	Valle de Bravo, Mexico State
PI 384063, pop2	18.83 N, -100.16 W	Mexico, Mexico State
PI 384071, pop3	18.33 N, -100.31 W	Iguala, Guerrero State
PI 566692, pop4	19.06 N, -100.41 W	Zitacuaro, Michoacán State
Ames 19288, Oh43	NA	Ohio United States
PI 550473, B73	NA	Iowa United States
NSL 30053, W22	NA	Wisconsin United States
PI 558532, Mo17	NA	Missouri United States
