# Supplementary material for: The potential role of genetic assimilation during maize domestication
Source: PLoS One. 2017 Sep 8;12(9):e0184202. doi: 10.1371/journal.pone.0184202 (PMC5590903; doi:10.1371/journal.pone.0184202)
Supplement: S2 Table — (PDF) [file pone.0184202.s002.pdf]

Table S2. Teosinte phenotypes in 2013 experiment.

All vegetative biomass (g)		Plant height (cm)		# Nodes	
**EHC**	**MCC**	**EHC**	**MCC**	**EHC**	**MCC**
241.2 ± 114.9	265.3 ± 95.8	141.8 ± 57.6	239.9 ± 48.3	15 ± 4.7	22 ± 4.5
