# Supplementary material for: The potential role of genetic assimilation during maize domestication
Source: PLoS One. 2017 Sep 8;12(9):e0184202. doi: 10.1371/journal.pone.0184202 (PMC5590903; doi:10.1371/journal.pone.0184202)
Supplement: S3 Table — (PDF) [file pone.0184202.s003.pdf]

Table S3. Maize phenotypes in 2014 experiment.

Vegetative biomass (g)		Cobs (g)		Total Biomass (g)		Height (cm)	
**EHC**	**MCC**	**EHC**	**MCC**	**EHC**	**MCC**	**EHC**	**MCC**
55.0 ± 18.3	69.5 ± 17.0	39.3 ± 14.9	64.5 ± 11.7	94.3 ± 32.6	134.0 ± 25.1	140.6 ± 19.3	176.4 ± 15.5
