# Supplementary material for: The potential role of genetic assimilation during maize domestication
Source: PLoS One. 2017 Sep 8;12(9):e0184202. doi: 10.1371/journal.pone.0184202 (PMC5590903; doi:10.1371/journal.pone.0184202)
Supplement: S4 Table — Plants from a maternal source with a maize-like phenotype in a previous experiment are marked. (PDF) [file pone.0184202.s004.pdf]

Table S4. Number of reads per sample. Plants from a maternal source with a maize-like phenotype in a previous experiment are marked.

Sample	Reads	Maize-like mom
265ppm_pop1.A.1	8643790	X
265ppm_pop1.A_2.1	7648528	
265ppm_pop1.B.1	5031069	
265ppm_pop2.A.1	6732943	
265ppm_pop2.B.1	5063618	X
265ppm_pop2.B_2.1	6467938	
265ppm_pop3.A.1	4399187	
265ppm_pop3.B.1	5564170	
265ppm_pop3.C.1	4139946	X
265ppm_pop4.A.1	7427625	
265ppm_pop4.B.1	3814875	
265ppm_pop4.C.1	4746629	X
400ppm_pop1.A.1	4070011	X
400ppm_pop1.A_2.1	5989185	
400ppm_pop1.B.1	7570271	
400ppm_pop2.A.1	7882249	
400ppm_pop2.B.1	5812095	X
400ppm_pop2.B_2.1	7455893	
400ppm_pop3.A.1	8630267	
400ppm_pop3.B.1	5433424	
400ppm_pop3.C.1	5029499	X
400ppm_pop4.A.1	5857687	
400ppm_pop4.C.1	8836575	X
265ppm_Oh43.A	14416915	
265ppm_Oh43.B	13978009	
265ppm_B73.A	15911016	
265ppm_B73.B	14202484	
265ppm_W22.A	16932095	
265ppm_W22.B	15442245	
265ppm_Mo17.A	14819357	
265ppm_Mo17.B	14669370	
400ppm_Oh43.A	14114563	
400ppm_Oh43.B	14914449	
400ppm_B73.A	20128181	
400ppm_B73.B	18545881	
400ppm_W22.A	16442158	
400ppm_W22.B	15726969	
400ppm_Mo17.A	16940428	
400ppm_Mo17.B	17291888	
