# Supplementary material for: The potential role of genetic assimilation during maize domestication
Source: PLoS One. 2017 Sep 8;12(9):e0184202. doi: 10.1371/journal.pone.0184202 (PMC5590903; doi:10.1371/journal.pone.0184202)
Supplement: S1 Fig — The teosinte plant in the EH chamber is a maize-like phenotype in vegetative architecture, inflorescence sexuality, and seed maturation, as described in the main text. The plant in the MA chamber is typical of teosinte today in those characteristics. These traits are unaltered for the maize plant between the EH chamber on the left and the MA chamber on the right. (PDF) [file pone.0184202.s005.pdf]

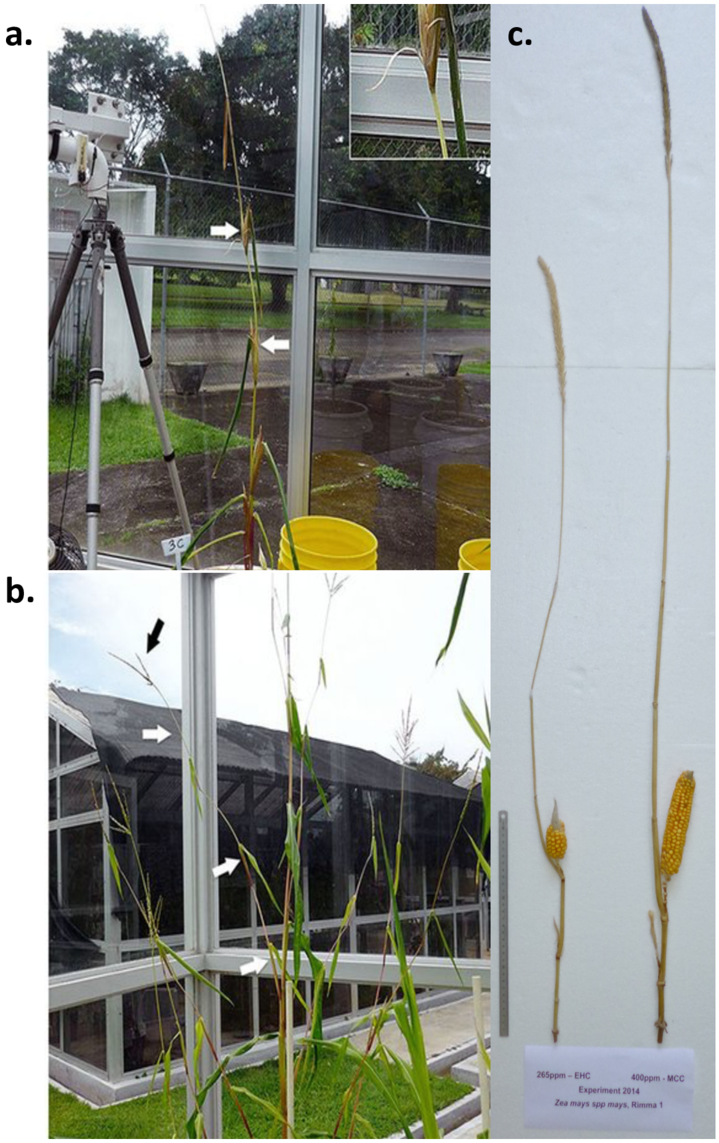

Fig S1. Examples of phenotypic differences in EH chamber on the top and MA chamber on the bottom for teosinte ((a.) and (b.) from Piperno and coauthors [26]) and maize (c.). The teosinte plant in the EH chamber is a maize-like phenotype in vegetative architecture, inflorescence sexuality, and seed maturation, as described in the main text. The plant in the MA chamber is typical of teosinte today in those characteristics. These traits are unaltered for the maize plant between the EH chamber on the left and the MA chamber on the right.
