# Supplementary material for: The potential role of genetic assimilation during maize domestication
Source: PLoS One. 2017 Sep 8;12(9):e0184202. doi: 10.1371/journal.pone.0184202 (PMC5590903; doi:10.1371/journal.pone.0184202)
Supplement: S2 Fig — (PDF) [file pone.0184202.s006.pdf]

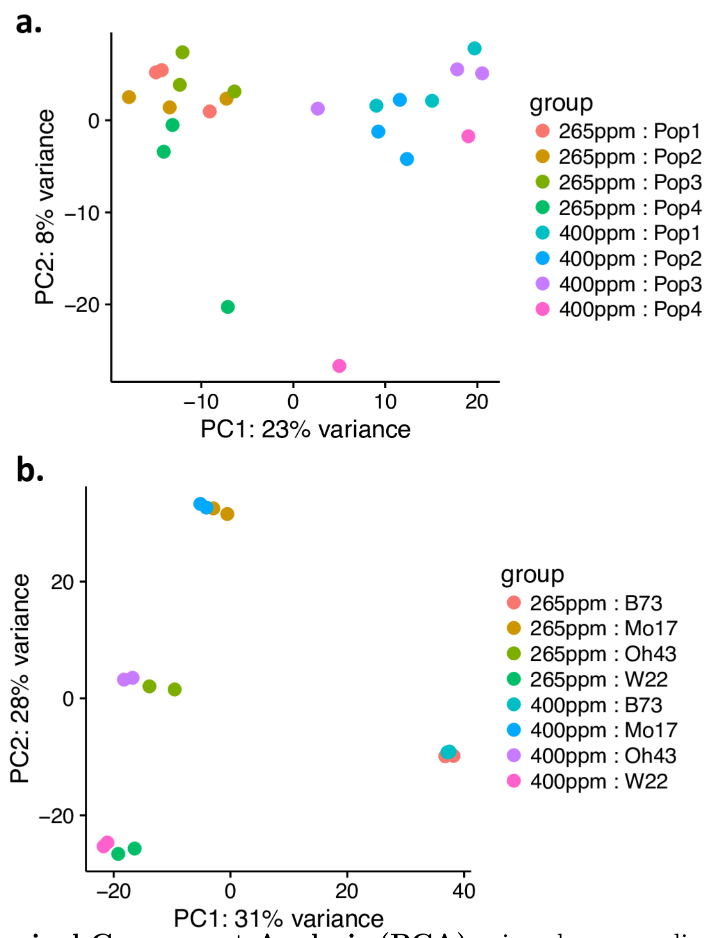

Fig S2. Principal Component Analysis (PCA) using rlog-normalized of the expression data for the principal components 1 (PC1) and PC2, for teosinte (a.) and maize (b.).
