# Supplementary material for: The potential role of genetic assimilation during maize domestication
Source: PLoS One. 2017 Sep 8;12(9):e0184202. doi: 10.1371/journal.pone.0184202 (PMC5590903; doi:10.1371/journal.pone.0184202)
Supplement: S3 Fig — Mann-Whitney U tests for all comparisons are significant (***P <0.001). (PDF) [file pone.0184202.s007.pdf]

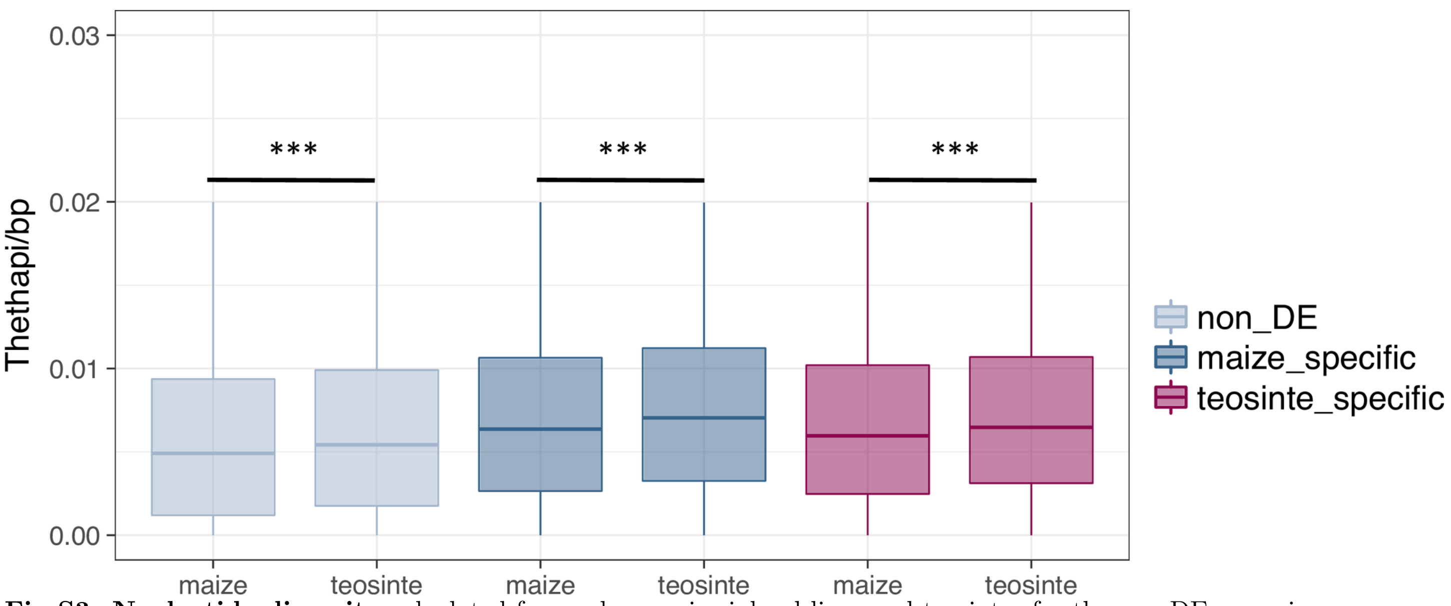

Fig S3. Nucleotide diversity calculated for modern maize inbred lines and teosintes for the non-DE genes in gray, maize-specific genes in blue and the teosinte-specific DE genes in red. Mann-Whitney U tests for all comparisons are significant (***P $<$0.001)
